# Distinguishing of Histopathological Staging Features of H-E Stained Human cSCC by Microscopical Multispectral Imaging

**DOI:** 10.3390/bios14100467

**Published:** 2024-09-29

**Authors:** Rujuan Wu, Jiayi Yang, Qi Chen, Changxing Yang, Qianqian Ge, Danni Rui, Huazhong Xiang, Dawei Zhang, Cheng Wang, Xiaoqing Zhao

**Affiliations:** 1 Institute of Biomedical Optics and Optometry, Key Lab of Optical Instruments and Equipment for Medical Engineering, Ministry of Education, University of Shanghai for Science and Technology, Shanghai 200093, China; wrj1226613633@163.com (R.W.); yangchangxing1998@163.com (C.Y.); qianqiange2021@163.com (Q.G.); dannie13887380564@163.com (D.R.); xiang3845242@163.com (H.X.); 2 Department of Dermatology, School of Medicine, Ruijin Hospital, Shanghai Jiao Tong University, Shanghai 200093, China; 18721961088@163.com; 3 Department of Phototherapy, Shanghai Skin Disease Hospital, School of Medicine, Tongji University, Shanghai 200093, China; chenqi235533@163.com; 4 Engineering Research Center of Optical Instruments and Systems, Ministry of Education, Key Laboratory of Modern Optical Systems, Shanghai University of Technology, Shanghai 200093, China; usstoe@vip.163.com

**Keywords:** microscopical multispectral imaging, cSCC, tumor staging

## Abstract

Cutaneous squamous cell carcinoma (cSCC) is the second most common malignant skin tumor. Early and precise diagnosis of tumor staging is crucial for long-term outcomes. While pathological diagnosis has traditionally served as the gold standard, the assessment of differentiation levels heavily depends on subjective judgments. Therefore, how to improve the diagnosis accuracy and objectivity of pathologists has become an urgent problem to be solved. We used multispectral imaging (MSI) to enhance tumor classification. The hematoxylin and eosin (H&E) stained cSCC slides were from Shanghai Ruijin Hospital. Scale-invariant feature transform was applied to multispectral images for image stitching, while the adaptive threshold segmentation method and random forest segmentation method were used for image segmentation, respectively. Synthetic pseudo-color images effectively highlight tissue differences. Quantitative analysis confirms significant variation in the nuclear area between normal and cSCC tissues (*p* < 0.001), supported by an AUC of 1 in ROC analysis. The AUC within cSCC tissues is 0.57. Further study shows higher nuclear atypia in poorly differentiated cSCC tissues compared to well-differentiated cSCC (*p* < 0.001), also with an AUC of 1. Lastly, well differentiated cSCC tissues show more and larger keratin pearls. These results have shown that combined MSI with imaging processing techniques will improve H&E stained human cSCC diagnosis accuracy, and it will be well utilized to distinguish histopathological staging features.

## 1. Introduction

Cutaneous squamous cell carcinoma (cSCC) is the second most prevalent malignant skin tumor, ranging from 20% to 50% [1,2]. It occurs due to the malignant proliferation of keratinocytes in the epidermis and could potentially invade surrounding tissues or metastasize [3]. cSCC is typically caused by factors such as exposure to ultraviolet (UV) radiation from the sun, chronic skin inflammation, immunosuppression, and certain genetic conditions [4]. Recently, the incidence and mortality rate of cSCC has been increasing worldwide due to the aging and growing population [5]. The disease-specific death rate resulting from metastatic cSCC was estimated to be between 1.5 and 2 per 100,000 person-years [6]. Early diagnosis and accurate staging of tumors hold immense value in the effective management of cSCC and further improving long-term outcomes.

Pathological diagnosis has traditionally been considered the gold standard, relying on the assessment of tissue structure and cytological features. The grading of cSCC is determined based on the extent to which the characteristics of squamous epithelium, such as intracellular bridges and keratinization, as well as pleomorphism and mitotic activity, can be effectively recognized [7,8]. Pathologically poorly differentiated cSCC plays a significant role in identifying high-risk cSCC, leading to unfavorable prognosis and necessitating more aggressive treatment measures. On the contrary, well differentiated cSCC serves as a crucial indicator for low-risk cSCC, associated with a favorable prognosis [9]. However, the assessment of differentiation levels (well or poorly) in pathological analysis relies predominantly on subjective judgments, therefore resulting in potential intra- and inter-observer variability. Consequently, there is a need for a valuable approach to overcome these limitations.

Biological tissues are usually non-homogeneous media. The form of light propagation in biological tissues changes as the composition and structure of biological tissues change. In addition, as the waveband changes, the way light interacts with biological tissues shows a significant diversity. Multispectral imaging (MSI) can generate a series of spectral images by capturing the same scene at multiple wavelengths across the spectrum [10]. The main difference between MSI and ordinary imaging technology is that it can obtain a high-resolution spectrum of each pixel of each image, rather than just the red, blue, and green images seen by the naked eye. MSI possesses a distinct capability for characterizing skin due to its ability to leverage the spatial relationships among different tissue absorption spectra within a local region. By employing spectral data cube analysis, complex spectral–spatial models can be employed, leading to more accurate classification of image features specific to targeted diseases [11]. Compared to traditional RGB color images, MSI provides more comprehensive spectral color information, making it particularly valuable in the analysis of histopathological images [12]. By leveraging the spectral characteristics and spatial information present in the multi-band image, the samples can be analyzed effectively.

Previous research conducted by Cheng et al. demonstrated the effective use of MSI in analyzing the pathology of cSCC in mice. Both qualitative and quantitative characteristics derived from MSI can be effectively utilized to comprehensively ascertain the malignancy of tissues. This research carries substantial implications for the advancement of a cost-effective and easy-to-operate device that not only simplifies pathological diagnosis but also enables convenient digital staining and access to crucial histological information [13]. Additionally, MSI was utilized for the analysis of fine needle aspiration smears in thyroid nodule detection by Shah et al. Their findings revealed that compared to traditional image analysis methods, applying watershed image segmentation to cells using MSI substantially reduced the occurrence of false positives [14]. Bautista et al. leveraged the spectral information from multispectral images to digitally dye sections and, in combination with MASSON staining, highlighted tissue fiber structures without the need for additional dyeing procedures [15], thereby emphasizing structures that were previously underappreciated [16]. Spreinat et al. established a near-infrared multispectral absorption imaging device to investigate the potential for tumor recognition in histological samples of melanoma, nodular basal cell carcinoma, and cSCC in the spectral range of 900 to 1500 nm [17].

The application of MSI allows for the exploration of tumor identification in human histopathology using existing pathological sections. However, it is crucial to acknowledge the existing limitation regarding a more precise classification of tumor pathology in cSCC. Therefore, in this study, we conducted routine pathological analysis of human cSCC and normal tissues using MSI. The primary objective of this paper is to bridge this gap by specifically investigating the identification and classification of tumor staging through MSI, providing valuable insights and contributing to advancements in this field.

## 2. Materials and Methods

### 2.1. Experimental Equipment

A microscopic MSI system was developed from previous studies, utilizing narrow-band light-emitting diode (LED) illumination. The system comprises three key modules: the light source module, the coupling module, and the imaging module. The light source module consists of 13 narrow-band LEDs with a spectral range from 420 nm to 680 nm and a bandwidth of 20 nm. It can be controlled by computer commands. The emitted light from the LEDs is shaped into a parallel beam through fiber and a coupling lens in the coupling module. This parallel beam is then directed into the imaging module. Within the imaging module, the beam is reflected by a spectroscope and subsequently illuminated by the objective. The light reflected by the sample passes through the objective once more before being focused onto the CMOS camera by the tube lens. The control program facilitates exposure and image acquisition for each LED, resulting in a 13-channel spectral image. The real-time adjustment function of the camera’s exposure time and gain parameters is realized by means of a slider control. There are specific performance parameters of the system in the literature [13].

### 2.2. Experimental Samples

The cSCC H&E stained slides were from the Shanghai Jiaotong University-affiliated Ruijin Hospital. Informed consent was obtained from all subjects or their legal guardian. The diagnosis and tumor stage were evaluated by two pathologists. All methods were carried out in accordance with relevant guidelines and regulations. All the relevant experimental protocols were approved by the Ethics Committee of the Shanghai Jiaotong University-affiliated Ruijin Hospital.

### 2.3. Data Collection

To ensure precise and consistent image acquisition, all capturing procedures are conducted within a controlled darkroom environment. The process is carried out under the supervision of two experienced pathologists. A total of 13 distinct gray image bands are collected for each lesion area. The mechanical platform translation gradually captures different sections of the slide, ensuring overlapping areas for subsequent image stitching. The exposure time of the images is meticulously controlled to maintain consistency under the illumination of the same waveband light source. Each histological sample has 5 slides. To reduce the impact of random noise, five images are acquired for each area, which reduces the error caused by one acquisition. An average processing strategy is employed, calculating the average gray level of five images within the same area. Furthermore, the adaptive two-dimensional gamma function method is used to correct any uneven illumination in the images, with the specific processing procedure referenced in the literature [13].

### 2.4. Image Stitching

**Figure 1:**
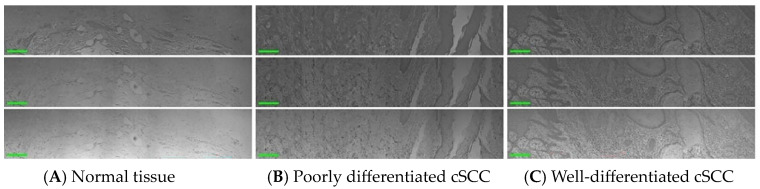
Single-band images of stained sections of human normal and cSCC slides at 520 nm, 600 nm, and 630 nm (scale bar 200 μm). (**A**) Normal skin tissue. (**B**) Poorly differentiated cSCC tissue. (**C**) Well-differentiated cSCC tissue.

Observations from single-field images are limited and prone to misjudgment and omission. Image stitching, on the other hand, expands the field of view, provides more comprehensive information, eliminates image borders, and achieves higher-resolution images. Therefore, this study conducted image stitching. Scale-invariant feature transform (SIFT) is employed to extract feature points from the images. Subsequently, the extracted feature points are matched to identify corresponding points across different images. The matched feature point pairs are utilized for image registration, ensuring precise alignment of all images. This step involves meticulous translation, rotation, and scaling to maintain spatial consistency. The registered image is then fused, followed by cropping of the edges and removal of stitching marks to create the final stitched image. The outcomes of this procedure are presented in Figure 1, depicting the single band images of various areas. These areas include the normal tissue section of the dermis without lesions and the cancerous area of the epidermis with poor- and well-differentiation. The selected wave bands for images are 520 nm, 600 nm, and 630 nm, respectively.

### 2.5. Statistical Analysis

The measurement values of the nucleocytoplasmic ratio are expressed as the mean, maximum, and minimum. To compare between two groups, Student’s t-tests were conducted. We used GraphPad Prism 9 software from GraphPad Software for analysis. Statistical significance was defined as *p* < 0.05.

## 3. Results

### 3.1. Human cSCC Manifestation in Pathology

**Figure 2:**
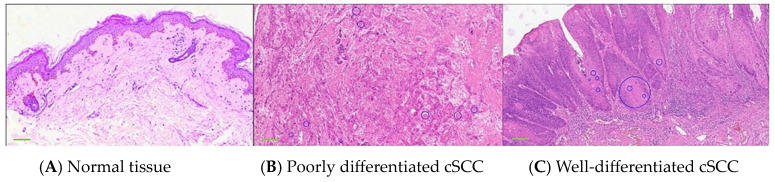
RGB images of normal skin tissue and cSCC histologically stained pathological sections. (**A**) Normal skin. (**B**) Poorly differentiated cSCC. (**C**) Well-differentiated cSCC. Blue circles represent keratin pearls, which are a typical qualitative feature distinguishing normal tissue from cSCC. The keratin pearls in the figure are marked circled by two specialized dermatological pathologists. The scale of a 4× image obtained by a 4× magnification microscope is 400 μm.

The pathological examination revealed distinct pathological alterations associated with cSCC, which originates from keratinocytes. It can be categorized into three subtypes based on the degree of keratinization: well-differentiated, moderately differentiated, and poorly differentiated. In this study, our focus is primarily on well-differentiated and poorly differentiated cases. In well-differentiated cSCC, the tumor cells demonstrate a classic squamous morphology characterized by distinct intercellular bridges and clear evidence of squamous differentiation. Mitotic figures are rare, reflecting the slower proliferation rate of these cells. Additionally, there is the presence of cells with incomplete or poor keratinization, along with the formation of keratin pearls, which are hallmark features of well-differentiated cSCC. In contrast, poorly differentiated cSCC exhibits a more aggressive and disorganized architecture. The tumor cells are arranged chaotically, with significant pleomorphism and a higher density of atypical cells, indicative of increased cellular dysregulation. Mitotic figures are more frequent, reflecting a higher proliferation rate. Furthermore, keratinization is either absent or minimal, lacking the well-formed keratin pearls seen in better-differentiated counterparts. These features highlight the more malignant behavior of poorly differentiated cSCC, often correlating with a worse prognosis and a higher potential for invasion and metastasis. Figure 2 exhibits the pathological image of normal skin and cSCC.

### 3.2. Features of Human cSCC Were Highlighted by an MSI System

To minimize discrepancies arising from conventional staining methods, this study utilized an MSI system equipped with LED illumination, encompassing 13 wavebands. This approach enables the utilization of spectral dimensions for precise representation of morphological characteristics specific to different tissue types.

Through the examination of the RGB images and multispectral images of both normal tissues and cSCC, we observed that multispectral imaging offers a superior capability in highlighting specific pathological features with enhanced clarity. This advantage stems from the capacity of multispectral imaging to selectively capture features across various wavebands, in contrast to RGB images, which may render such features with diminished visibility. Moreover, multispectral images across different wavelengths exhibit distinct imaging effects on various tissue components. These distinctions facilitate a more precise differentiation between normal tissue, poorly differentiated cSCC, and well-differentiated cSCC. Therefore, multispectral analysis enables more effective extraction of differences in cellular morphology among normal tissue, poorly differentiated cSCC, and well-differentiated cSCC.

In addition, the pathological sections of cSCC after Hematoxylin eosin staining (H&E) were used in this study. H&E staining is the most widely used staining method in pathology and histology. Its dyeing principle is the chemical bond between the tissue and the dye. Because different components of tissues or cells have different affinity for dyes and different staining properties, hematoxylin components stain the nucleus blue-black, with good nuclear details, while eosin stains the cytoplasm and most connective tissue fibers with different hues and intensities of pink, orange, and red. They have unique optical properties that allow them to help pathologists effectively distinguish some cellular structures under the microscope [18]. The absorption peak of hematoxylin is about 560 nm, but there is also significant absorption in the 400–600 nm range. Eosin’s absorption peaks are about 518 nm and 528 nm, but it is absorbed in the 450–650 nm range [19]. The 520 nm band is close to a major absorption peak of eosin, and eosin-stained collagen fibers and diseased epidermal tissues will appear particularly clear because this band can capture the staining of these structures well, so as to effectively distinguish between normal and cancerous tissues. The 600 nm band is close to the subabsorption peak of eosin and has a good response to both chromatin and cytoplasm. Large fat droplets in normal tissue and keratinized beads and squamous pits in cancerous tissue will appear more pronounced. This is because the absorption properties of eosin in this band give these structures a high contrast in multispectral images. Moreover, 630 nm is in another significant absorption band of eosin, also close to the absorption range of hematoxylin, and the hematoxylin-stained nucleus has significant absorption properties in this band, thus making the nucleus prominent in the image. Therefore, using the three bands of 520 nm, 600 nm, and 630 nm for multispectral imaging can make full use of the optical properties of hematoxylin and eosin in H&E staining.

**Figure 3:**
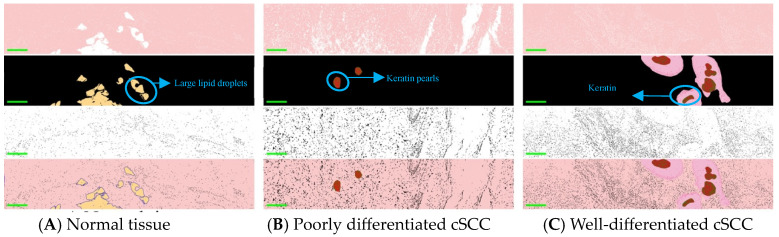
The images are arranged in four rows. The segmented pseudo-color images from top to bottom correspond to 520 nm, 600 nm, and 630 nm, respectively, followed by the segmented composite image (with a scale of 200 μm). The three columns of images from left to right are (**A**) Normal skin tissue. (**B**) Poorly differentiated cSCC tissue. (**C**) Well-differentiated cSCC tissue. Among them, the yellow part in A represents large lipid droplets in normal tissue, the red part in B and C represents keratin pearls in cSCC, and the pink part in C represents squamous pits in well-differentiated cSCC.

Figure 3 shows the distinctive structural features of normal skin and cSCC in single-wave band and pseudo-color composite images. In images across different bands, the grayscale values of highlighted structures change significantly. Three bands of 520 nm, 600 nm, and 630 nm were selected for image segmentation. For the 520 nm and 600 nm wavebands, an adaptive threshold segmentation method was used, while for the 630 nm waveband, a random forest method was applied for segmentation. Specifically, the images at the 520 nm waveband were used for the segmentation of collagen fibers in normal tissue and diseased epidermis in cancerous tissue. The images at the 600 nm waveband were primarily used to segment large lipid droplets in normal tissues and keratin pearls in cancer tissues. The images at the 630 nm waveband were primarily used to segment the cellular nucleus. Keratosis was more obvious in well-differentiated cSCCs compared to poorly differentiated ones. The mixed image of pseudo-color not only qualitatively distinguishes normal skin, poorly and well-differentiated tissue but also mitigates the impact of staining differences on diagnostics, offering pathologists a more objective and accurate diagnostic basis.

### 3.3. Differentiation between Normal Skin and cSCC Was Accomplished through Nucleo-Plasmic Ratio

A high nucleo/plasmic ratio serves as a crucial indicator of tumor development and carcinogenesis, for nucleus density is elevated in cancer cells. In order to make a more accurate statistical analysis of the nucleo/cytoplasm ratio, the sliding window technique was used in this study. Specifically, a 200 × 200 pixels size window was created to search for areas of interest in the image. Two image stitchings were performed on each slice. Five 200 × 200 windows were captured on one spliced image. Therefore, a total of 10 200 × 200 windows were collected on each pathological section. During the search, a threshold limit was imposed on the selection to ensure that only those areas with appropriate exposure were selected. In addition, the center of gravity in the window was determined to exclude the area of under-exposure or the junction between the nucleus and the keratin pearls. Subsequently, the nucleo/plasmic ratio was calculated within the automatically selected five windows.

**Figure 4:**
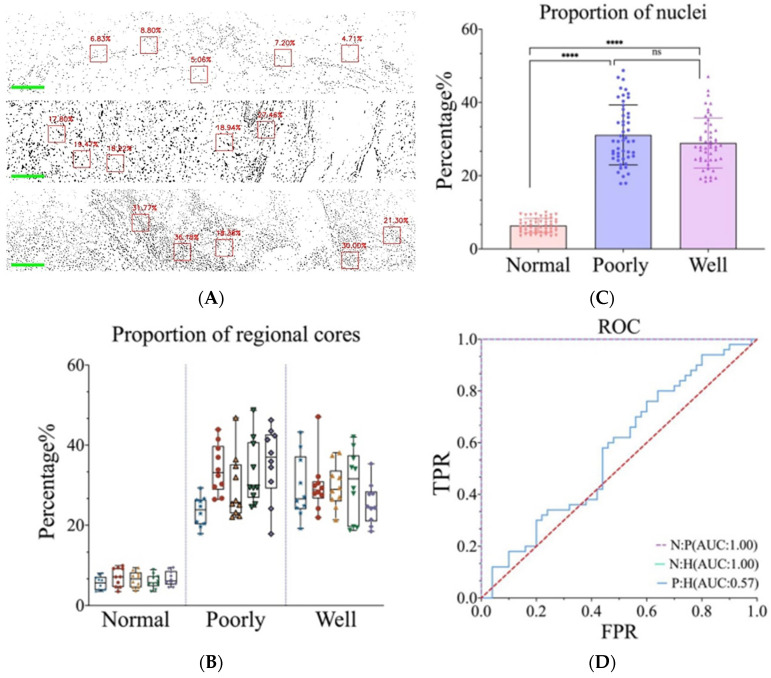
(**A**) Nucleo/plasmic ratio at each window of normal skin, poorly differentiated cSCC, and well-differentiated cSCC. (**B**) Distribution of the nucleo/plasmic ratio of skin tissue cells in each image. Different colors represent different images. (**C**) Nucleo/plasmic ratio statistical data of normal skin, poorly and well-differentiated cSCC. **** represents the *p* value. (**D**) ROC curve of recognition rate. (Normal tissues: Poorly differentiated cSCC; Normal tissues: Well-differentiated cSCC; Poorly differentiated cSCC: Well-differentiated cSCC).

As shown in Figure 4A, the mosaic images depict the nucleo/cytoplasmic ratio per window of normal, well-differentiated, and poorly differentiated tissues. The nucleo/plasmic ratio of normal skin is significantly lower compared to that of cSCC. Statistical analysis was performed on ten windows for each slice, and the distribution of the nucleo/cytoplasmic ratio is shown in Figure 4B. which also shows that there is a significant difference in nuclei area between normal skin and cSCC (*p* < 0.001) (Figure 4C). For further discrimination between these tissues, we conducted an in-depth analysis of the specificity and accuracy of the nuclear area proportion. The resultant ROC curve comparisons among normal, poorly differentiated, and highly differentiated tissues are displayed in Figure 4D.

### 3.4. Poorly and Well-Differentiated cSCC Was Identified via Nuclear Atypia and Keratin Pearls

Although it is possible to differentiate cSCC from normal skin based on the nucleo/plasmic ratio, this method does not provide discrimination between high and poor levels of differentiation. Therefore, within a single field of view, the examination of keratin pearls and the quantitative analysis of nuclear heterogeneity were investigated.

**Figure 5:**
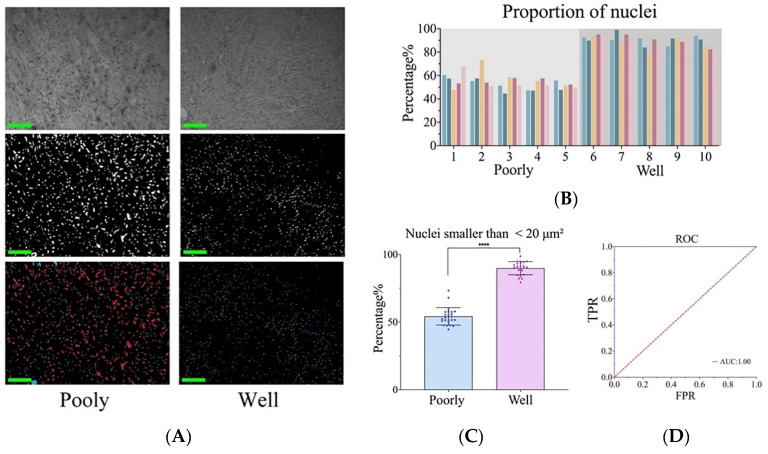
Nuclear atypia in cSCC. (**A**) From top to bottom, the images are presented in the following order: original image, segmented image, and pseudo-color image. Nuclei smaller than 20 μm^2^ in blue, nuclei larger than 20 μm^2^ in red. (**B**) Proportion of cell nuclei with an area smaller than 20 μm^2^ in each image. (**C**) Nucleo/plasmic ratio statistical data of poorly and well-differentiated cSCC. **** represents the *p* value. (**D**) ROC curve for recognition rate.

The nuclear size was calculated within segmented individual nuclear images as a measure of nuclear heterogeneity. The proportion of nuclei below 10 μm^2^, 20 μm^2^, 30 μm^2^, and 40 μm^2^ relative to the total number of nuclei in the segmentation image were calculated from five fields of section on each slide. The analysis revealed that utilizing a threshold of 20 μm^2^ effectively distinguished between poorly and well-differentiated cSCC. All nuclei exceeding 20 μm^2^ were distinctly marked on the segmentation image (Figure 5A). In the subsequent data analysis, five images were evaluated for each specimen slide. We calculated the proportion of cell nuclei having an area smaller than 20 μm^2^ in each image. The percentage of nuclei smaller than 20 μm^2^ in poorly differentiated cSCC was significantly lower than that in well-differentiated tissues (*p* < 0.001) (Figure 5B,C). Then, we conducted specificity and accuracy analyses to thoroughly evaluate the number and proportion of nuclei, and the results of the ROC curve are shown in Figure 5D.

**Figure 6:**
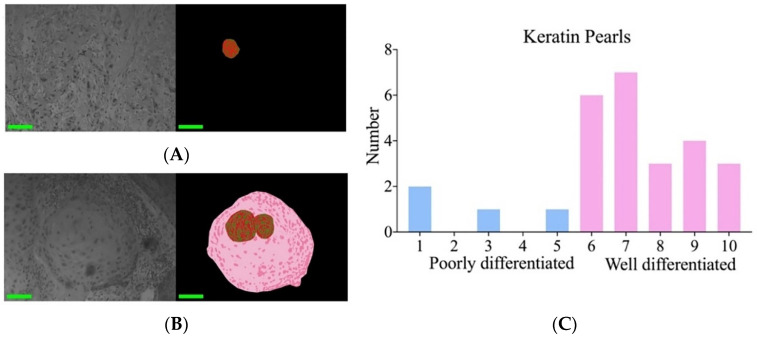
Keratin pearls in cSCC. (**A**) Original image and segmented pseudo-color image of poorly differentiated tissue. (**B**) Original image and segmented pseudo-color image of well-differentiated tissue. (**C**) The number of keratin pearls in the upper 10 images per slide was calculated.

In this study, we also found that poorly and well-differentiated cSCC could be distinguished by keratin pearls. By quantifying ten non-contiguous regions on each slide, the results indicated that in well-differentiated cSCC, the area of keratin pearls was significantly larger and more abundant compared to poorly differentiated samples. Figure 6A,B presents the relevant pseudo-color diagram depicting segmentation. Figure 6C presents the number of keratin pearls within ten windows for each specimen slide. Slides 1 through 5 are categorized as poorly differentiated, while slides 6 through 10 are classified as well-differentiated.

## 4. Discussion

Histopathology is vital for assessing perineural infiltration, tumor differentiation, and tumor depth, all of which significantly impact tumor stage determination and prognosis [20]. According to National Comprehensive Cancer Network (NCCN) criteria, cSCC stage is classified into high-risk and low-risk categories [21]. Low-risk cSCC should be histopathologically moderate or well-differentiated, which demonstrates less aggressive behavior. A retrospective study encompassing 136 patients with aggressive cSCC located in the trunk or limbs, conducted at a tertiary care center between 1994 and 2004, revealed that poorly differentiated tumors had a threefold increased risk of metastasis or death compared to well or moderately differentiated tumors [22,23]. It is estimated that the probability of metastasis in poorly differentiated tumors ranges from 33% to 58% [24]. Thus, the influence of pathological stage on cSCC tumor staging is a crucial aspect to consider, especially for early diagnosis.

However, the diagnosis of cSCC is relatively straightforward. The challenge lies in accurately differentiating between well and poorly differentiated, as this greatly affects tumor staging and patient prognosis [25]. According to the World Health Organization (WHO), intercellular bridges and keratinization serve as crucial diagnostic criteria for squamous epithelium and can be discerned during the initial stages of cSCC [7]. In this study, it was first proposed that MSI was used to differentiate well-differentiated cSCC from poorly differentiated cSCC by analyzing the nuclear atypia and the number of keratin pearls, avoiding potential visual inconsistencies among pathologists.

Compared to hyperspectral imaging (HSI), MSI is simpler in terms of data acquisition and processing, particularly when aiming to reduce complexity, save time, and lower costs. In addition, whether the absorption, scattering, or fluorescence of interaction between light with biological tissue, the spectra curves are continuous with almost no separated spectral lines. MSI has demonstrated sufficient accuracy in the current experiment, especially in distinguishing between normal and diseased tissues, and strikes a balance between performance and cost. Therefore, in clinical practice, MSI may be more practical due to its ease of use and lower implementation costs compared to HSI. However, HSI technology, with its higher spectral resolution and richer spectral information, enables more precise classification and diagnostic outcomes. HSI has shown great potential in clinical oncology, especially in monitoring tissue perfusion during treatment. For example, HSI has demonstrated significant promise in assessing flap perfusion after breast reconstruction and anastomotic perfusion during gastrointestinal reconstruction, offering key perfusion data in a non-contact, non-invasive manner [26]. In recent years, the clinical applications of HSI have been concentrated in the gastrointestinal field, particularly in assessing anastomotic perfusion and identifying tumor margins. Studies by Jansen-Winkeln et al. have shown that HSI, combined with neural network algorithms, can achieve high sensitivity and specificity in tumor margin classification [27]. Additionally, their research confirmed that HSI can rapidly and accurately differentiate between well-perfused and poorly-perfused tissue regions. The combination of HSI and fluorescence imaging with indocyanine green has also proven to provide complementary information in identifying perfusion boundaries, enhancing diagnostic accuracy [28]. Studier-Fischer et al. also used HSI to capture spectral data of kidney tissue and analyzed this data with artificial intelligence to monitor intraoperative renal perfusion in real-time, providing surgeons with a non-invasive assessment of renal blood flow and oxygenation [29]. While HSI offers significant advantages in some advanced applications, the MSI system adopted in our study offers a more practical solution that meets the research objectives. Therefore, although MSI is sufficient in certain cases, the potential value of HSI, particularly its advantages in complex clinical environments, should not be overlooked and deserves greater attention in future research and applications.

There have been various strategies employed to characterize tissues from a histological perspective. MSI is a rapidly growing field with broad applications in both preclinical and clinical contexts. It involves the combined utilization of imaging and spectroscopy, thus offering a synergistic approach to understanding biological phenomena [30]. In contrast to conventional RGB full-color imaging, MSI enables the acquisition of 3-dimensional datasets or a cube (x, y, and λ). These datasets can be further utilized for quantitative imaging of individual analytes after proper calibration. Habibalahi et al. demonstrated the potential of autofluorescence multispectral imaging (AFMI) to differentiate between ocular surface squamous neoplasia (OSSN) and pterygium by leveraging the fluorescence spectral signatures of intrinsic cellular compounds, notably protoporphyrin IX, NADH, and FAD [12]. However, the implementation of AFMI is accompanied by various challenges. The intrinsic biological heterogeneity among patients often results in variations in fluorescence characteristics, presenting challenges in achieving consistent diagnosis. The depth of penetration remains a limitation for AFMI, as it could not capture the complexities of deeper tissue strata effectively, resulting in overlooking significant lesions or tumors [31]. Moreover, the presence of auto-fluorescence from peripheral tissues or non-target cells can introduce the possibility of false positives, emphasizing the importance of meticulous examination and scrutiny.

In current pathology practice, chromogenic immunohistochemistry (IHC) is utilized [32]. However, far more advanced and powerful approaches are available, such as multiplexed IHC (mIHC). These techniques offer great insights into examining disease heterogeneity and characterizing underlying biological mechanisms that drive disease. Additionally, mIHC could help conserve limited resources. One notable benefit of mIHC is enhanced accuracy through image analysis facilitated by landmark markers that indicate tissue architecture. These markers can also accelerate scan times. This workflow not only increases a pathologist’s productivity by automating the measuring of parameters that are difficult to reliably achieve visually but also emphasizes the role of the pathologist in reviewing analysis results as an integral part of the workflow. Though mIHC provides profound insight into molecular cascades while preserving tissue context, interpreting multiplexed stained samples can be difficult in current practice. The utilization of fluorescence in mIHC often leads to the blending of multiple targets, which has the potential to obscure visual assessment. Moreover, formalin-fixed and paraffin-embedded tissues could exhibit tissue autofluorescence, further complicating visual interpretation [33].

Pathological biopsy continues to be the established standard for diagnosis, with a focus on enhancing diagnostic efficiency and addressing discrepancies caused by staining variations. This study aims to classify the histopathological staging features of human cSCC using multispectral imaging. The research demonstrated the application of this method for the diagnosis and tumor staging of cSCC and highlighted its ability to provide both qualitative and quantitative data. Notably, the pathological slices used in this study were conventional H&E-stained slices, requiring no further processing. These findings validate the clinical application capabilities of our custom-designed multispectral microscopic imaging system. By incorporating techniques such as image stitching, digital staining human cSCC tissue sections, and segmentation of the key pathological features in tumor stages, this system enhances visual clarity and simplifies the diagnostic process. Such technology holds promise for reducing equipment costs and minimizing the personnel required for pathological diagnosis.

Our future research direction involves analyzing the intrinsic spectral characteristics of a broader range of tissue elements in skin diseases using both image and spectral approaches. We will also explore advanced feature extraction algorithms to extract typical pathological features from spectral or feature channel images. Additionally, our aim is to achieve digital staining on unstained sections, further advancing system automation and artificial intelligence. By collecting more clinical data and accumulating multispectral data from various diseases, we anticipate providing more efficient and high-quality diagnostic solutions.

In conclusion, our study successfully showcased the application of MSI for the diagnosis and staging of cSCC. This system enhances visual clarity by effectively distinguishing between well-differentiated and poorly differentiated tissues based on crucial histopathological staging features, including the nucleo/plasmic ratio, area of keratin pearls, and proportion of cell nuclei. These advancements not only reduce the need for personnel in pathological diagnosis but also offer valuable insights and lay a foundation for advancing system automation and artificial intelligence in this field.

## Data Availability

All data generated or analyzed during this study are included in this published article.
